# Whale counting in satellite and aerial images with deep learning

**DOI:** 10.1038/s41598-019-50795-9

**Published:** 2019-10-03

**Authors:** Emilio Guirado, Siham Tabik, Marga L. Rivas, Domingo Alcaraz-Segura, Francisco Herrera

**Affiliations:** 1 Andalusian Research Institute in Data Science and Computational Intelligence, University of Granada, 18071 Granada, Spain; 2 Andalusian Center for the Assessment and Monitoring of Global Change, University of Almería, 04120 Almería, Spain; 3 Department of Biology, University Marta Abreu of Las Villas, 50100 Santa Clara, Cuba; 4 iecolab. Interuniversity Institute for Earth System Research, University of Granada, 18071 Granada, Spain

**Keywords:** Machine learning, Marine mammals

## Abstract

Despite their interest and threat status, the number of whales in world’s oceans remains highly uncertain. Whales detection is normally carried out from costly sighting surveys, acoustic surveys or through high-resolution images. Since deep convolutional neural networks (CNNs) are achieving great performance in several computer vision tasks, here we propose a robust and generalizable CNN-based system for automatically detecting and counting whales in satellite and aerial images based on open data and tools. In particular, we designed a two-step whale counting approach, where the first CNN finds the input images with whale presence, and the second CNN locates and counts each whale in those images. A test of the system on Google Earth images in ten global whale-watching hotspots achieved a performance (F1-measure) of 81% in detecting and 94% in counting whales. Combining these two steps increased accuracy by 36% compared to a baseline detection model alone. Applying this cost-effective method worldwide could contribute to the assessment of whale populations to guide conservation actions. Free and global access to high-resolution imagery for conservation purposes would boost this process.

## Introduction

Whales, which comprise some of the largest animals that have ever existed, have always thrilled humans^1,2^. Whales had and keep an enormous economic and societal value^3^. More than 13 million whale-watchers were registered in 2008 across 119 countries, generating a global economic activity of US$ 2.1 billion^4^. Since whales generally are long-living species at high trophic-levels, they play an essential role to structure marine food webs, and to maintain ecosystem functions and services^5,6^. In the past, commercial whaling depleted whale populations from 66% to 90% from their original numbers, which subsequently caused alterations in marine biodiversity and functions^7^. To prevent whales from extinction, the signatories of the International Convention for the Regulation of Whaling limited whale hunting to scientific or aboriginal actions since 1946, although the moratorium on commercial whaling did not come into force until 1982^8^. Even though, there still exist a great uncertainty around the number of whales in the oceans and the viability of their populations^9^. In late 2017, the Species Red List of the International Union for Conservation of Nature (IUCN)^10^ reported that 22% of 89 evaluated cetacean species were classified as threatened, whereas almost 50% species could not be evaluated due to the lack of data. Hence, a more accurate estimation of whale distribution and population sizes is essential to warrant cetacean conservation^11^.

The process of identifying and estimating the number of cetaceans is normally carried out^12,13^ (1) *in situ*, from ships^14,15^, planes^16,17^ or ground stations^18^, by using visual surveys^19,20^, acoustic methods^21,22^, or a combination of both^23,24^; or (2) e*x situ*, by using satellite tracking^25,26^ or photo-interpretation or classical image classification techniques on Very High Resolution (VHR) aerial or satellite images^27–31^. However, these methods are costly, not robust against scenario changes (e.g., different regions or atmospheric conditions), not generalizable to a massive set of images, and often require handcrafted features^32,33^. Indeed, biodiversity conservation would certainly benefit from robust and automatic systems to assess species distributions, abundances and movements from satellite and aerial images^34,35^.

Deep learning methods, particularly Convolutional Neural Networks (CNNs), could help in this sense since CNNs are already outperforming humans in visual tasks such as image classification and object detection^36^. CNN models have the capacity to automatically learn the distinctive features of different object classes from a large number of annotated images to later make correct predictions on new images^37^. Although the construction of a dataset for training is costly, the learning of CNNs on small datasets can be boosted by data-augmentation, which consists of increasing the volume of the training dataset artificially, and additionally by transfer learning, which consists of starting the learning of the network from a prior knowledge rather from scratch^38,39^.

Identifying whales from aerial and satellite images using CNNs at a global scale is very challenging for several reasons: (1) comprehensive datasets with VHR images of whales to train CNNs do not exist yet; (2) VHR images are expensive and relatively scarce in the marine environment; (3) whales could potentially be confused with other objects such as boats, rocks, waves, or foam; (4) whale postures or behaviour captured in a snapshot are quite variable since different parts of whale bodies can be emerged or submerged (e.g. blowing, logging, etc.); and (5) occlusions and noise could occur due to clouds, aerosols, haze, sunglint, or water turbidity.

In this work, we propose a large scale generalizable deep learning system for automatically counting whales from satellite and aerial images. For this, we combined two CNNs into a two-step model, where the first CNN detects the presence of whales and the second CNN counts the number of whales in the images (See Methods section). To overcome the above mentioned challenges, (1) we combined several open datasets to build an annotated training database of high quality vertical images of whales and of objects that could be confused with whales, (2) we used data augmentation and transfer learning techniques to make the CNNs robust to image variability, (3) we assessed the effect of whale posture and location on the model performance, and, as a proof of concept, (4) we applied the model to free Google Earth coastal imagery in 10 whale-watching hotspots. Additionally, we compared the performance of our combined approach to the performance reached just using the second CNN alone.

## Contributions

The main contributions of this work can be summarized as follows:

- It presents the first proof of concept on how deep learning can be exploited for counting whales in RGB aerial and satellite images and using free machine learning software.
- It addresses the problem of whale counting at large scale areas by using a two-step approach: (1) the first step CNN selects the candidate images with a high probability of whale presence, and (2) the second step CNN analyzes these images by a detection model to localize and count the existing whales. Combining these two steps increased accuracy by 36% compared to the baseline detection model alone.
- It provides two datasets that guarantee a good learning for the first-step and second-step CNN-based models, with 2,100 images. For the external evaluation, this work also provides a new test dataset made of 13,348 images of ten marine mammal hotspots.
- It analyzes the effect of whale postures or behavior on model performance.
- It provides evidence on how a CNN based system trained on higher resolution aerial images of whales is able to find whales in lower resolution satellite images.

### Preliminaries on CNN models for image classification and object detection in images

Deep Neural Networks (DNNs) are a subset of machine learning algorithms able to learn from a training dataset to make predictions on new examples called testset. They are built using a hierarchical architecture of increasing sophistication, each level of this hierarchy is called layer. One of their main particularities is their capacity to extract the existent features from data automatically without the need of external hand crafted features. Under the supervised learning paradigm, DNNs provide a powerful framework when trained on a large number of labelled samples.

Convolutional Neural Networks (CNNs) are a specialized type of neural networks capable of extracting spatial patterns from images. Their architecture is built by staking three main types of layers: (1) convolutional layer, which is used to extract features at different levels of the hierarchy, (2) pooling layer, which is essentially a reduction operation used to increase the abstraction level of the extracted features and (3) fully connected layer, which is used as a classifier at the end of the pipeline.

CNNs need a large number of examples to achieve good learning. However, building dataset from scratch is costly and time consuming. To overcome these limitations in practice, two techniques are used: Transfer learning and data-augmentation. Transfer-learning consists of using the knowledge acquired in problem A to problem B. This is implemented by initializing the weights of the model for problem B using the pre-trained weights on problem A. Data-augmentation consists of applying specific transformations to the training images. In general, these transformations simulate the deformations that data could suffer in real world, e.g., scaling, rotations, translations, different illumination conditions, cropping parts of the image. It was demonstrated in several works that data-augmentation increases the robustness and generalization capacity of CNNs^40^.

CNNs constitute the state-of-the art in all the fundamental tasks in computer vision, e.g., in image classification and object detection in images. In image classification, the CNN model has to analyze the input image and produce a label that describes its visual content, together with a probability that expresses the confidence of the model. In object detection, the CNN detection model has not only to produce the correct label but also determine by means of a bounding box the region in the input image where the target object is located. Examples of the most accurate and robust models for image classification are Inception^41^ and Inception ResNet^42^. The most accurate detection frameworks are end-to-end object detection models that combine a sophisticated detection technique with one of the most powerful CNN classification models. At present, there exist several detection frameworks that provide good trade-off between accuracy, robustness and speed, such as, Faster-RCNN^36^, YOLO9000^43^, FPN^44^, RefineDet^45^, DSSD^46^ and Focal Dense Object Detection^47^. Furthermore, several studies are focusing on improving these frameworks on specific remote sensing data^48–51^. In this work, we used Faster-RCNN^36^ based on Inception RenNet v2, as it is the most accurate detection framework according to the this study^48^.

## Results

### Whale presence detection model (step-1) validation

**Figure 1:**
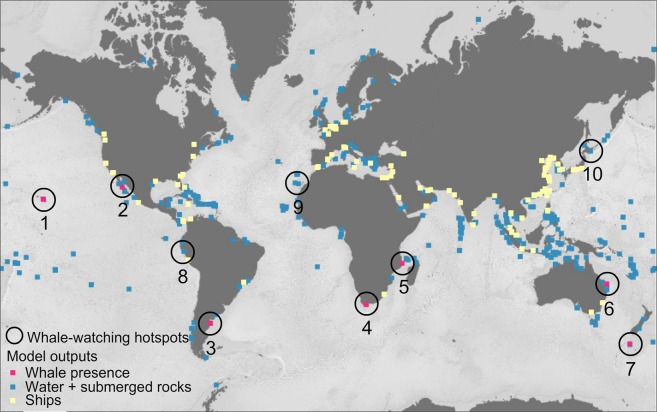
Results at a global scale of the first step whale presence detection model in ten marine mammal hotspots for whale watching (details in Table 1). Red, blue, and yellow cells indicate respectively whale presence, water + submerged rocks, and ships.

**Figure 2:**
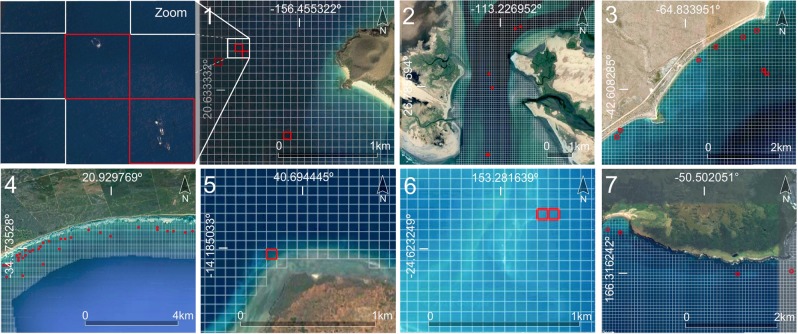
Illustration of the assessed grid cells where the first step CNN-based model detects presence of whales. The cells with whale presence are indicated in red boxes in six of the ten candidate hotspots. In the three remaining hotspots, high resolution images were not available for the whale watching months. Map data: Google, DigitalGlobe.

The analysis of the first step CNN-based model on ten marine mammal hotspots for whale watching (Fig. 1) confirmed the presence of whales in six of the ten assessed whale watching hotspots (Fig. 2). The acquisition dates of the satellite images available through Google Earth for these six sites matched the known whale watching period from the literature (Tables 1, 2). In the whale watching hotspot located in Memba (Mozambique), the spatial resolution of Google Earth images was not sufficient for the human annotator to determine with a high confidence whale presence and hence, the prediction of the whale presence model was tagged as uncertain. In the three sites where the model did not find whales (Peru, Canary Islands, and Japan), the acquisition date of their Google Earth images was not within the known whale watching period but during the migration season. In the Peruvian coast and in the Canary Islands the detection was particularly challenging since the images presented rough sea.

**Table 1:** Summary of the results of step-1 CNN-model in a total number of 13,348 evaluated cells.

Site IDs Site names (country)	Cells with whales (photo-interpreted)	Cells with whales (CNN-based model)	Total number of cells
1. Hawaiian Islands (USA)	4	4	565
2. Baja California (Mexico)	7	6	2,974
3. Valdés Peninsula (Argentina)	7	4	1,295
4. Witsand (South Africa)	40	35	3,312
5. Memba (Mozambique)*	U	1	594
6. Coral Sea (Australia)	2	2	346
7. Enderby Island (New Zealand)	8	4	790
8. Peruvian coast (Peru)	0	0	1,307
9. Canary Islands (Spain)	0	0	1,045
10. Japanese coast (Japan)	0	0	1,120
Total	68	56	13,348

The first column shows the IDs and names of the ten evaluated whale watching sites. The second column shows the number of cells with whale presence photo-interpreted by the authors. The third and fourth columns show respectively the number of cells with and without whales according to step-1 CNN-based model predictions in each site. The asterisk and U indicates labeling uncertainty due to the low resolution of the images in that region.

**Table 2:** Sources and characteristics of the images used for the external verification of the whale presence detection model (step-1).

Site IDs Site names (country)	Latitude Longitude WGS84	Whale watching period	Date of Google Earth image	Season of acquisition date	Image source Pixel size (m)
1. Hawaiian Islands (USA)	20.636602, −156.462511	Dec-Apr^89^	Apr 3, 2013 Jan 13, 2013	Breeding	USGS aerial 0.15 M
2. Baja California (Mexico)	26.769961, −113.242382	Feb^90^	Feb 20, 2017	Breeding	WV-03 0.31 P 1.24 M
3. Valdés Peninsula (Argentina)	−42.603384, −64.810850	May-Dec^1^	Sep 18, 2003	Breeding	QB-02 0.61 P 2.5 M
4. Witsand (South Africa)	−34.390203, 20.879985	Jul-Oct^4^	Aug 9, 2009	Breeding	GE-01 0.46 P 1.84 M
5. Memba (Mozambique)	−14.185282, 40.691405	Jun-Jul^91^	Jun 23, 2017	Breeding	SPOT-6 1.5 P 6.0 M
6. Coral Sea (Australia)	−24.622170, 153.291559	Sep-Nov^92^	Sep 13, 2005	Breeding	QB-02 0.61 P 2.5 M
7. Enderby Island (New Zealand)	−50.501698, 166.282294	Jul-Sep^93^	Sep 2, 2012	Breeding	WV-02 0.46 P 1.84 M
8. Peruvian coast (Peru)	−14.253483, −76.159243	Jun-Sep^94^	Mar 9, 2016	Migrating/Feeding	WV-03 0.3 P 1.24 M
9. Canary Islands (Spain)	28.139039, −16.796631	Aug-Nov^95^	Mar 10, 2017	Migrating	WV-02 0.46 P 1.84 M
10. Japanese coast (Japan)	41.947425, 143.246413	Apr^96^	Oct 5, 2014	Migrating	WV-02 0.46 P 1.84 M

Location of the ten evaluated sites. Match between the known whale watching period from the literature and the acquisition date and season of the satellite images in Google Earth. For each image source in Google Earth (reduced spectral resolution) these metadata are provided: the satellite (GE-01: GeoEye-01; QB-02: QuickBird-2; SPOT-6; USGS: United States Geological Survey orthoimagery; WV-02: WorldView-2; and WV-03: WorldView-3), the pixel size at nadir in m, and the sensor spectral resolution (M: Multispectral; P: Panchromatic).

**Figure 3:**
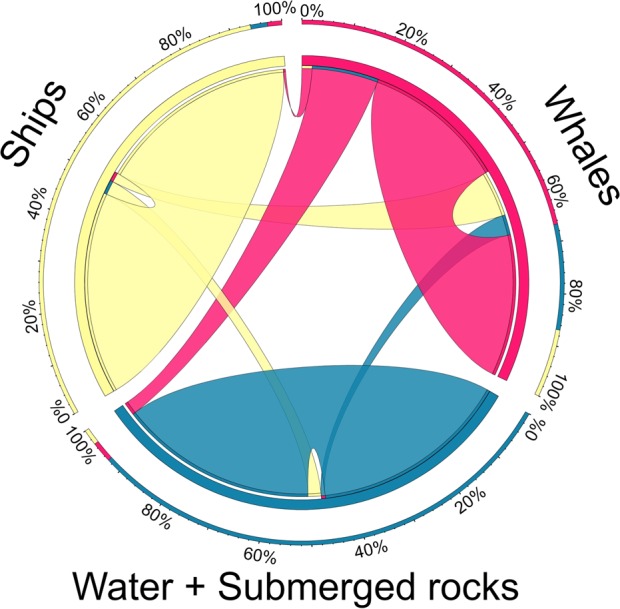
Visualization (Circos plot) of the confusion matrix between the photo-interpreted ground truth and the predictions made by the CNN-based model (step-1) for detecting the presence of whales (in red), ships (yellow), and water + submerged rocks (blue). The links between classes depicts false negatives (whales that were misclassified as ship or water + submerged rock) and false positives (ships or water + submerged rocks that were misclassified as whales), the thickness of these links indicate the percentage of misclassified instances. Errors and successes are shown as a percentage on the outer concentric bars. Only 13 and 1 whale images were classified respectively as water + submerged rocks and as ships, while only 9 ship images and 4 water + submerged rocks images were classified as whales.

Step-1 CNN-based model that detects the presence of whales reaches an average F1-measure of 81.8% for whales, 95.9% for water + submerged rocks and 96.7% for ships (Table S2). Only 20.58% of test grid cells containing whales were misclassified as water (19.11%) or ships (1.47%). A very small number of water + submerged rocks and ship images were misclassified as whales (1.00% and 2.25%, respectively; see Fig. 3). An example of a false positive that shows a hand-glider over the sea in Witsand (South Africa) is illustrated in (Fig. S2).

**Figure 4:**
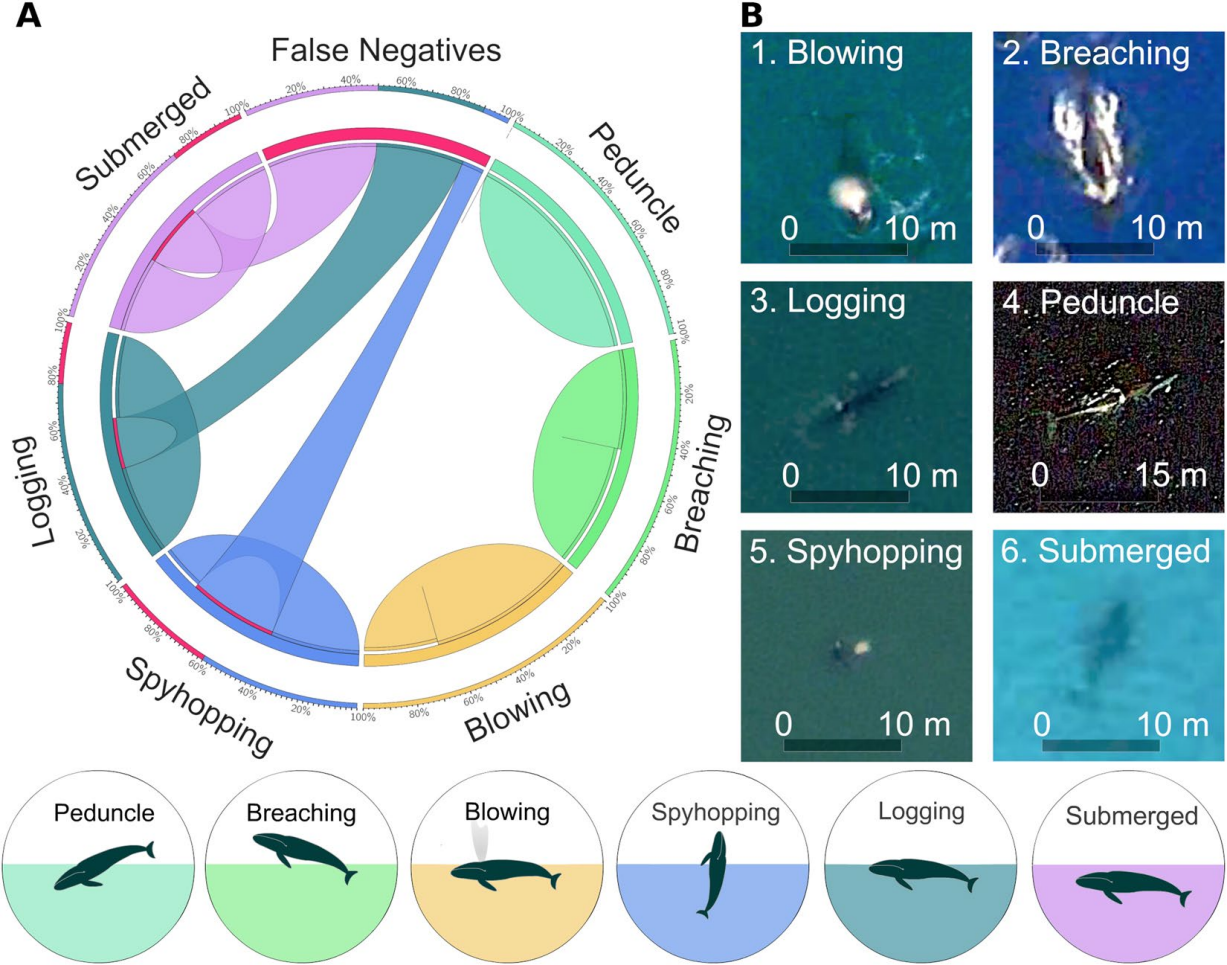
Impact of whale postures or behaviour on the performance of the step-1 CNN-based model for detecting the presence of whales. (**A**) The Circos plot shows the distribution of the false negatives (undetected whales, in red color) and true positives across whale postures. Whales under blowing, breaching, and peduncle postures were better detected than under spyhopping, logging and submerged. (**B**) Example of images for each behaviour from the detected hotspots at the highest zoom. Map data: Google, DigitalGlobe.

Whales behaviour affects the performance of the first step CNN-based model for detecting the presence of whales (Fig. 3). Higher detectability (greater than 90% of true positives) was obtained for the following whale postures: blowing, breaching, peduncle, and logging. The lowest detectability occurred for submerged and spyhopping postures (33% and 60% of false negative, respectively; see Fig. 4A). Indeed, the lower performance of step-1 model in the Argentinean and New Zealand sites (Table S1, Fig. S1) was due to the much greater frequency of these latter postures in the images (see Data S5). Overall, greater number of whales were in the passive behaviour of logging and submerged (60% of detected whales and 74% of photo-interpreted whales), while the lower number of whales were detected under active movements (Fig. 4A, Data S5).

### Whale counting (step-2) model validation

**Figure 5:**
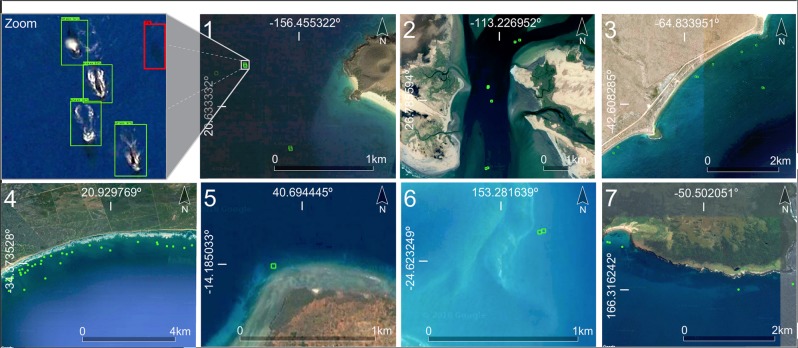
The results of the whale counting (step-2) CNN-based model that locates and counts the number of whales (green bounding boxes) in the grid cells in which step-1 CNN gave high probability for whale presence. The red bounding box shows a false negative. Map data: Google, DigitalGlobe.

The second step CNN-based model for localizing and counting whales analyzes only the cells where step-1 found whale presence (Fig. 5). From a total number of 84 whales photo-interpreted in this study across six hotspots for whale watching around the world, step-2 automatically localized and counted 62 of them, which gives the model an overall performance of 94% ± 0.015% of F1 measure (Table S1 and Fig. S3).

## Discussion and Conclusions

This study illustrates how global cetacean conservation could benefit from the operational application of deep learning on VHR satellite images. Using a two-step convolutional neural network model trained with a reduced dataset and applied on free Google Earth imagery, we managed to automatically detect and count 62 whales in six hotspots for whale watching around the world, reaching an overall global F1-measure of 78% ± 0.07% (F1 measure of 81% ± 0.13% for presence detection and 94% ± 0.01% for locating and counting). Our results show how the acquisition date of the satellite image, the behaviour recorded in the image and the resolution of the image can influence whale presence detection and counting. For instance, the spatial resolution of SPOT-6 satellite images was not good enough to assess whether the model was correct in Memba (Mozambique) at the date and location chosen. This robust, transparent and automatic method can have direct and wide implications for whale conservation by assessing whale distributions, abundances, and movement behaviours globally from satellite and aerial images.

Our satellite and aerial based assessment can complement and be compared with other aerial, marine, and land observations. The coastal images of Google Earth at zoom 18 that we used correspond to a visual altitude of ~254 m, similar to the aerial surveys for grey whales, and up to ~4 km offshore the coast, the maximum distance for whale visual surveys from land^52^. In whale assessments, such distances are good enough to get reliable estimates of instantaneous presence and relative population abundances^53^. As new RGB images become available, our method also enables dynamic updates at low cost, to assess seasonal and interannual changes in population sizes, feeding and breeding areas, migratory routes, and distribution ranges around the world.

Several studies show that the performance of CNNs can be equal or even better than humans when the quality of the images is good, for instance, for skin cancer detection^54^, mastering the game of Go^55^, or generalizing past experiences to new situations^56^. In general, the quality of the images determines the accuracy of the classification in CNNs^57^, learning and performing better on higher resolution images^58^. However, our results show how CNN-based methods trained on high-quality images (see methods section) can also reach good performance in classification and detection on medium-quality images, such as those available for free in Google Earth. In addition, the CNN-based models are robust^59^ against the differences in spatial and illumination angles across the different satellite sensors used in Google Earth^38^. Automatic image classification methods with convolutional neural networks can save time with respect to manual visual image classification methods^60^. In addition, human fatigue conditions the efficiency of labeling images^61^.

The use of free Google Earth imagery is convenient but it also has limitations since these are RGB images rather than multispectral, only available for few dates that may not be within the known whale presence period, are generally constrained to limited locations along coastal areas (up to ~4 km offshore), and are restricted for massive access. These last three limitations must be overcome together with the use of supercomputing for the worldwide “wall-to-wall” application of this method but do not impede its use for local assessments of whales around the world. Image spatial resolution can also limit the application of this method to detect cetaceans shorter than 5 m long (e.g. pilot whales, dolphins, etc.), which would require pixel sizes smaller than 1 m. For example, in our study, higher resolution images tend to give higher F1-measure (Table S3), though low contrast between whales and surrounding water tend to decrease performance (e.g. New Zealand) and high contrast to improve it (Table S3).

Our results showed that the behaviour and the image acquisition date can also bias the probability of detecting whales. The spatial pattern of whales under blowing, breaching, and peduncle postures showed better detectability than under logging and submerged, when whale bodies can be confused with submerged rocks and seafloor. However, the greater number of whales (both detected by the model and photo-interpreted) in our study were under passive (logging and submerged) instead of active behaviour, and in images captured during the breeding season. Therefore, the best time to identify whales might be along the breeding season (Table 1), when whales spend more time in surface and in shallow waters^62^. The effect of overlapped positions between females and calves on their detectability and counting should be further studied. In contrast, the most difficult time might be during migration and in the feeding season (Table 1), when whales are mainly in spyhopping, peduncle, and deeply submerged postures^63^, and in areas with low contrast between water and whales, or under high sea surface roughness, sea glint, or bad atmospheric conditions (clouds or aerosols).

The application of CNNs in remote sensing opens a world of possibilities for biodiversity science and conservation^64,65^. The great performance obtained by the CNN-based models trained on and applied to free VHR images opens the possibility to automatically process millions of satellite images around the world from whale hotspots, marine protected areas, whale sanctuaries, or migratory routes. Our procedure requires less time and lower cost than the traditional acoustic surveys from ships or the visual surveys from planes and helicopters. The efficiency of remote sensing methods is particularly relevant to save time and money for long-term whale monitoring in remote places, or under difficult circumstances such as whales trapped inside sea ice in polar regions^66^. The detection of whales using satellite images was already achieved using classical methods^29^, but their portability to other regions or dates was strongly limited by the necessity of spectral normalization. However, our CNN-based model is easily transferable to any region or RGB image with different characteristics in color, lighting and atmospheric conditions, background, or size and shape of the target objects, and it requires no human supervision, which speeds up the detection process^37^.

Further research could increase the performance and variety of species identified by our CNN-model. For instance, the model could be improved by increasing the number of samples and variety of atmospheric and sea conditions in the training datasets, by building hierarchical training datasets with different behaviour across different species^67^, by using more spectral bands and temporal information^68^, and by artificially increasing the spatial resolution of the images through rendering^69^. In addition, as it is a fast and scalable method, it can even be transferred to very high spatial resolution images (<10 cm) captured by unmanned aerial vehicles (UAVs) for the automatic identification of specific individuals^70^.

A global operationalization of our satellite-based model for whale detection and counting could greatly complement traditional methods^12–22^ to assist whale conservation, to guide marine spatial planning^71^, or to assess regional^11^ and global^72^ priorities for marine biodiversity protection against global change^73^. In addition, our method could be extended to higher resolution RGB images in particular and VHR multispectral data in general to identify and quantify cetaceans species^35^ and other marine species such as seals and sea lions^74^, penguins^75^, etc. To boost this process, free access to satellite data is key^76^. The compromise with biodiversity conservation from corporations such as Google, Microsoft, Planet, Airbus, or DigitalGlobe^77^ could be materialized through the systemic release of free high resolution aerial and satellite imagery at least from key sites for marine conservation. Even more, the acquisition of these images in pelagic environments does not directly compete with satellites commercial activity, which is usually focused on terrestrial and coastal areas. Having these images available would also make it possible to organize the development of a global database of images of cetaceans and many other marine vertebrates that could be used to improve the training of our whale detection and counting model or to develop similar models for other marine organisms. Images of the highest spatial resolution (such as WorldView-3 satellite images with a pixel size of 0.3 m) are particularly appropriate for this purpose. This way, satellite and CNN-based detectors of big marine organisms could serve to produce global characterizations of species populations and traits and of community composition as part of the initiative by the Group on Earth Observations - Biodiversity Observation Network (GEOBON) on satellite remote sensing essential biodiversity variables^78^.

## Methods

We address the problem of whale counting in large scale areas represented by a large number of VHR satellite and airborne images using a two-step approach that combines two models: (i) an image classification model and (ii) a whale detection model. To build these models, we needed to build two training datasets, one for each model. In this section, we first present the proposed two-step approach for whale counting then describe the process we used for building the training and test datasets for each step. In addition, we compare the performance of our two-step approach with a baseline approach based only on the detection model (Faster RCNN).

### The proposed two-step approach

**Figure 6:**
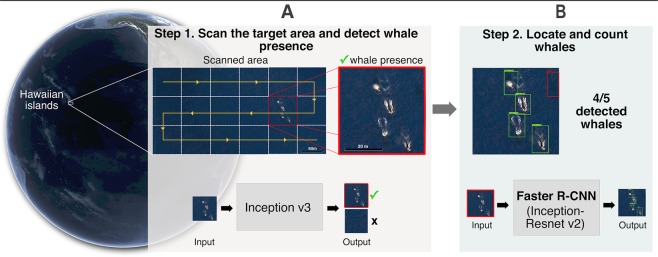
The proposed automatic whale-counting procedure with a two-step CNN-based model. (**A**) The first-step CNN scans the sample area (following the yellow line) to search for the presence of whales in each grid cell (white squares). Only grid cells in which the first-step CNN gives high probability for whale presence (red square) are analyzed by (**B**) the second-step CNN, which finally locates and counts individuals (the four green bounding boxes indicate correctly detected whales and the red box indicates a false negative). Map data: Google, DigitalGlobe.

Counting whales in large scale areas that can be represented by a large number of images is not only a complex task but also expensive from a computational point of view. To overcome these limitations, we propose a two-step CNN-based approach capable of counting whales in vast areas with a reduced computational cost, where the first CNN is used to filter out water potential false positives (ships, foam and rocks) but keeping candidate images to be analyzed later by the second and much slower CNN. To overcome these limitations, we designed a two-step whale counting approach, where the first and quicker CNN filters out images of just water or containing potential false positives (e.g., ships, foam, and rocks) but keeping input images with whale presence, and the second and slower CNN locates and counts each whale in the latter images. Thanks to this combination of two CNNs, our model is capable of counting whales in vast areas with a reduced computational cost. In our proof of concept, the first step CNN-based model analyzes 10 whale hotspots around the world represented by 13,348 grid-cells using a 71 × 71 m sliding window -twice the size of blue whales (30 m)- and outputs the probability of having detected whales in each cell (Fig. 6A). To reduce the computational cost of the overall approach, the second step CNN-based model analyzes only those cells with high probability of whale presence, localizes each whale within a bounding box, and outputs the number of counted individuals (Fig. 6B). On average, step-1 was less time consuming than step-2 by one order of magnitude (while step-1 only took around 1.02 seconds/image, step-2 took around 12.35 seconds/image, both in a laptop with a 1.6 GHz i5 CPU and 8 GB of RAM).

To facilitate its use and to support whale conservation, the CNN-based model was built using open-source software and can be used on free Google Earth images (subjected to terms of service). To increase the volume of the training dataset, we used data-augmentation techniques by applying rotation with a factor selected randomly between 0 and 360°, randomly flipping half of the training images, randomly cropping, random the scale size of the images, and random the brightness level of pixels by a factor of up to 50%.

We used Google TensorFlow deep-learning framework^79^ to train, validate and test the step-1 CNN-based model, and Google Tensorflow Object Detection API^80^ to train, validate and test the step-2 CNN-based model.

### Step-1: Whale presence detection phase

When seen from space, whales are often confused with other object classes such as ships and wave foam around partially or entirely submerged rocks. To give the first step CNN-based model the capacity to distinguish between these objects, we addressed the problem as a three-class image classification task. The first model was built using the last version of GoogleNet Inception v3 CNN architecture^41^, pretrained on the massive ImageNet database (around 1.28 million images, organized into 1,000 object categories). We retrained the parameters of the two last fully connected layers in the network on our dataset, using a learning rate of 0.001 and a decay factor of 16 every 30 epochs. As optimization algorithm, we used RMSProp with a momentum and decay of 0.9 and epsilon of 0.1.

To assess whether whale posture, season, and location affected whale presence detection in satellite images, we compared the F1-measure metric across different seasons and locations of the world, and across multiple active and resting behaviour^64^.

### Step-2: Whale counting phase

We built the second CNN-based model that counts whales by reformulating the problem into an object detection task. We used the detection model Faster R-CNN based on Inception-Resnet v2 CNN architecture^42,81^, pre-trained on the well known COCO (Common Objects in Context) detection dataset, which contains more than 200,000 images organized into 80 object categories^82^. The two last fully connected layers of the network were retrained on our dataset using a learning rate of 0.001 and a decay factor of 16 every 30 epochs. As optimization algorithm, we used RMSProp with a momentum of 0.9 and epsilon of 0.1.

### Training, testing and validating datasets

Currently, there does not exist any accessible datasets of satellite or aerial RGB images for whales detection. We had to build two datasets for training the CNN-based models to respectively detect the presence of whales and count their number, and a third dataset for testing and validating the whole procedure. We built the training datasets using satellite and aerial images of different resolutions so that the models can generalize correctly to different resolutions, contrasts and colors. The three datasets were built by combining, preprocessing and labeling images selected from the only sources available to us: Google Earth^38^, free Arkive^83^, NOAA Photo Library^84^, and NWPU-RESISC45 dataset^85^. For step-1, the training dataset contains 2,100 images of the following three classes (700 images per class): (1) whales, (2) ships, and (3) “water + submerged rocks” (Data S1). Whale images for training the CNN were mainly aerial images. For step-2, the training dataset contains 700 aerial images, with whales and background, in which each whale is annotated within a bounding box (the total number of bounding boxes is 945).

The dataset for testing and validating the whole procedure consists of RGB (Red, Green, and Blue bands) images downloaded from Google Earth in 14,148 cells of 71 × 71 m distributed worldwide. For ships, we selected 400 images from 100 seaports around the world (Data S2). For “water + submerged rocks” class, we selected 400 coastal images randomly around the world (Data S3). Finally, for whales (Table 1), we downloaded 13,348 cells (Data S4) of 71 m × 71 m from 10 areas that had very high-resolution images at zoom 18 (eye altitude of ~254 m) and that are known for marine mammal diversity or whale watching. These areas have been highlighted either as global marine biodiversity hotspots^86^, marine mammal hotspots^72^, irreplaceable or priority conservation sites (threshold >=0.3)^11^, and are included within or next to a marine protected area^87^ (Table 1). Two authors visually inspected all the images to annotate each cell with the name of the corresponding class and with the number of whales. From the 13,348 cells in the 10 hotspots for whale watching, the authors’ visual photo-interpretation revealed whales only in 68 cells.

The annotators also verified the presence of whales in these areas through specialized websites on whale watching and used the time-lapse tool of Google Earth to differentiate whales from sea floor and submerged rocks by comparing images from the same spot at different dates. Finally, to assess the effect of whale posture or behaviour on model performance, the annotators tagged each of the 68 cells with whale presence with the most dominant or conspicuous posture in it, by choosing from the following active and resting behaviour^88^, i.e., logging, breaching, spyhopping, blowing, peduncle, and submerged.

### Comparison between our two-step approach and the baseline detection model (Faster R-CNN)

For comparison purposes, we trained and analyzed Faster RCNN directly on the input images without any previous analysis. On the same test images (ten hotspots), Faster RCNN obtains an average F1 42%, which is substantially lower than the results obtained by our two-step approach (see Table S1D). This low performance is mainly due to the high number of false positives (e.g. boats, foam, rocks), specially in the sites with lower resolution images. Whereas, in site with very high resolution images, i.e., Hawaiian Islands (USA), Faster R-CNN obtained comparable results with our two-step approach, with F1 of 94%. The main reason why our two-step approach reaches much better accuracy than the detection model alone is that step-1 filters out most possible false positives, which consequently helps the next stage, step-2, in finding whales more accurately.

### Metrics used in the performance assessment

To evaluate the performance of both CNN-based models, we used these metrics^39^: positive predictive value, sensitivity, and F1-measure (Table 3).

**Table 3:** Accuracy indicator, equation, and interpretation of the performance assessment.

Accuracy indicator	Equation	Interpretation
Positive predictive value (or precision)	$\frac{truepositives}{truepositives+falsepositives}$	In how many images the assigned class was correct.
Sensitivity (or recall)	$\frac{truepositives}{truepositives+falsenegatives}$	From all images of a class, how many were correctly are detected.
F1-measure (or F1-score)	$2x\frac{positivepredictivevaluexsensitivity}{positivepredictivevalue+sensitivity}$	Index that evaluates the balance between precision and recall.

True positives correspond to images that were correctly classified or counted as whales by the models, false positives correspond to images that were classified or counted as whales by the models but actually corresponded to another class, and false negatives correspond to undetected images with whales. In simple terms, high positive predictive value means that the model returned substantially more actual whales than false ones, while high sensitivity means that the model returned most of the actual whales. F1-measure provides a balance between precision and sensitivity. We used 5-fold Cross-Validation strategy to evaluate our two-step approach and the baseline on the test dataset.

## Supplementary information

Supplementary Information

Supplementary Dataset 1

Supplementary Dataset 2

Supplementary Dataset 3

Supplementary Dataset 4

Supplementary Dataset 5

## Data Availability

The test and training datasets that support our findings are available from Github archive (https://github.com/EGuirado/CNN-Whales-from-Space). Restrictions apply to the availability of the images of the training and validation data, which were used with permission for the current study, but are not publicly available. Some data may be available from the authors upon reasonable request and under written permission from Google Earth, Arkive, or NOAA Photo Library when applicable. To allow reproducibility, in the Supplementary Information, we provide the metadata of all the images in the training and testing datasets.
