# Supplementary material for: Whale counting in satellite and aerial images with deep learning
Source: Sci Rep. 2019 Oct 3;9:14259. doi: 10.1038/s41598-019-50795-9 (PMC6776647; doi:10.1038/s41598-019-50795-9)
Supplement: Supplementary file 1 — Supplementary Information [file 41598_2019_50795_MOESM1_ESM.docx]

# **SUPPLEMENTARY INFORMATION for “Whale counting in satellite and aerial images with deep learning” by Emilio Guirado, Siham Tabik, Marga L. Rivas, Domingo Alcaraz-Segura, Francisco Herrera.**

**SUPPLEMENTARY TABLES**

Table S1. Performance of the two step CNN-based model in whale watching hotspots (13,348 71 m × 71 m assessed grid cells) for A) whale presence detection (step-1), B) whale counting (step-2), and C) the whole procedure. TP is True Positive, FP is False Positive, FN is False Negative, PPV is Positive Predictive Value, U is uncertain, and F1 is the F1-measure.

1. **Step-1, CNN-based model for whale presence identification by site**

| **Site ID, name and country** | **TP** | **FP** | **FN** | **PPV** | **Sensitivity** | **F1** |
| --- | --- | --- | --- | --- | --- | --- |
| 1. Hawaiian Islands (USA) | 4 | 0 | 0 | 1.00 | 1.00 | 1.00 |
| 2. Baja California (Mexico) | 6 | 2 | 1 | 0.75 | 0.86 | 0.80 |
| 3. Valdés Peninsula (Argentina) | 4 | 0 | 3 | 1.00 | 0.57 | 0.73 |
| 4. Witsand (South Africa) | 34 | 1 | 6 | 0.97 | 0.85 | 0.91 |
| 5. Memba (Mozambique) | U | U | U | - | - | - |
| 6. Coral Sea (Australia) | 2 | 1 | 0 | 0.67 | 1.00 | 0.80 |
| 7. Enderby Island (New Zealand) | 4 | 1 | 4 | 0.80 | 0.50 | 0.62 |
|  |  |  | **Total** | **0.86±0.14** | **0.80±0.21** | **0.81±0.13** |

**B) Step-2, CNN-based model for whale counting by site**

| **Site ID, name and country** | **TP** | **FP** | **FN** | **PPV** | **Sensitivity** | **F1** |
| --- | --- | --- | --- | --- | --- | --- |
| 1. Hawaiian Islands (USA) | 8 | 0 | 1 | 1.00 | 0.89 | 0.94 |
| 2. Baja California (Mexico) | 7 | 0 | 2 | 1.00 | 0.78 | 0.88 |
| 3. Valdés Peninsula (Argentina) | 4 | 0 | 0 | 1.00 | 1.00 | 1.00 |
| 4. Witsand (South Africa) | 37 | 3 | 4 | 0.93 | 0.90 | 0.91 |
| 5. Memba (Mozambique) | U | U | U | - | - | - |
| 6. Coral Sea (Australia) | 2 | 0 | 0 | 1.00 | 1.00 | 1.00 |
| 7. Enderby Island (New Zealand) | 4 | 0 | 1 | 1.00 | 0.80 | 0.89 |
|  |  |  | **Total** | **0.99±0.005** | **0.89±0.045** | **0.94±0.015** |

**C) Global CNN-based model for whale presence and counting by site**

| **Site ID, name and country** | **TP** | **FP** | **FN** | **PPV** | **Sensitivity** | **F1** |
| --- | --- | --- | --- | --- | --- | --- |
| 1. Hawaiian Islands (USA) | 8 | 0 | 1 | 1.00 | 0.89 | 0.94 |
| 2. Baja California (Mexico) | 7 | 2 | 2 | 0.78 | 0.78 | 0.78 |
| 3. Valdés Peninsula (Argentina) | 4 | 0 | 3 | 1.00 | 0.57 | 0.72 |
| 4. Witsand (South Africa) | 33 | 4 | 7 | 0.90 | 0.79 | 0.84 |
| 5. Memba (Mozambique) | U | U | U | - | - | - |
| 6. Coral Sea (Australia) | 2 | 1 | 0 | 0.67 | 1.00 | 0.80 |
| 7. Enderby Island (New Zealand) | 4 | 1 | 5 | 0.80 | 0.44 | 0.57 |
|  |  |  | **Total** | **0.86±0.08** | **0.74±0.20** | **0.78±0.07** |

**D) Only Faster RCNN model for whale detection and counting by site**

| **Site ID, name and country** | **TP** | **FP** | **FN** | **PPV** | **Sensitivity** | **F1** |
| --- | --- | --- | --- | --- | --- | --- |
| 1. Hawaiian Islands (USA) | 8 | 0 | 1 | 1.00 | 0.89 | 0.94 |
| 2. Baja California (Mexico) | 7 | 6 | 2 | 0.54 | 0.78 | 0.64 |
| 3. Valdés Peninsula (Argentina) | 4 | 236 | 3 | 0.02 | 0.57 | 0.03 |
| 4. Witsand (South Africa) | 37 | 719 | 4 | 0.05 | 0.90 | 0.09 |
| 5. Memba (Mozambique) | U | U | U | - | - | - |
| 6. Coral Sea (Australia) | 2 | 5 | 0 | 0.29 | 1.00 | 0.44 |
| 7. Enderby Island (New Zealand) | 4 | 7 | 5 | 0.36 | 0.44 | 0.40 |
|  |  |  | **Total** | **0.38±0.33** | **0.76±0.20** | **0.42±0.31** |

Table S2. Performance of the step-1 CNN-based model for all classes (Whale presence, Ships, and Water + submerged rocks) in all assessed grid cells throughout the world (71 m × 71 m grid cells assessed). TP is True Positive, FP is False Positive, FN is False Negative , PPV is Positive Predictive Value, and F1 is the F1-measure.

| **Class** | **TP** | **FP** | **FN** | **PPV** | **Sensitivity** | **F1** |
| --- | --- | --- | --- | --- | --- | --- |
| Whale presence | 54 | 10 | 14 | 0.84 | 0.79 | 0.818 |
| Ships | 378 | 2 | 22 | 0.99 | 0.95 | 0.967 |
| Water + submerged rocks | 396 | 30 | 4 | 0.93 | 0.99 | 0.959 |
| Total | 828 | 42 | 40 | 0.95 | 0.95 | 0.953 |

Table S3. Effect of image resolution, contrast between whales and water, and whale posture on the performance (F1-measure) of the step-1 CNN-based model for whale presence detection across the six sites assessed (sorted by increasing performance, F1-measure of step-1). The asterisk and U indicates labeling uncertainty due to the low resolution of the images in that region.

| **Site ID, name and country** | **F1 Step-1** | **panchromatic resolution**  **(m/pixel)** | **Behaviour in FN** | **Water-Whale contrast** |
| --- | --- | --- | --- | --- |
| 5. Memba (Mozambique)* | U | 1.50 | - | - |
| 7. Enderby Island (New Zealand) | 0.62 | 0.46 | 50% submerged  50% logging | low |
| 3. Valdés Peninsula (Argentina) | 0.73 | 0.61 | 100% submerged | low |
| 6. Coral Sea (Australia) | 0.80 | 0.61 | - | high |
| 2. Baja California (Mexico) | 0.80 | 0.31 | 100% submerged | medium |
| 4. Witsand (South Africa) | 0.91 | 0.46 | 66% submerged  33% spyhopping | low |
| 1. Hawaiian Islands (USA) | 1.00 | 0.15 | - | medium |

**SUPPLEMENTARY FIGURES**


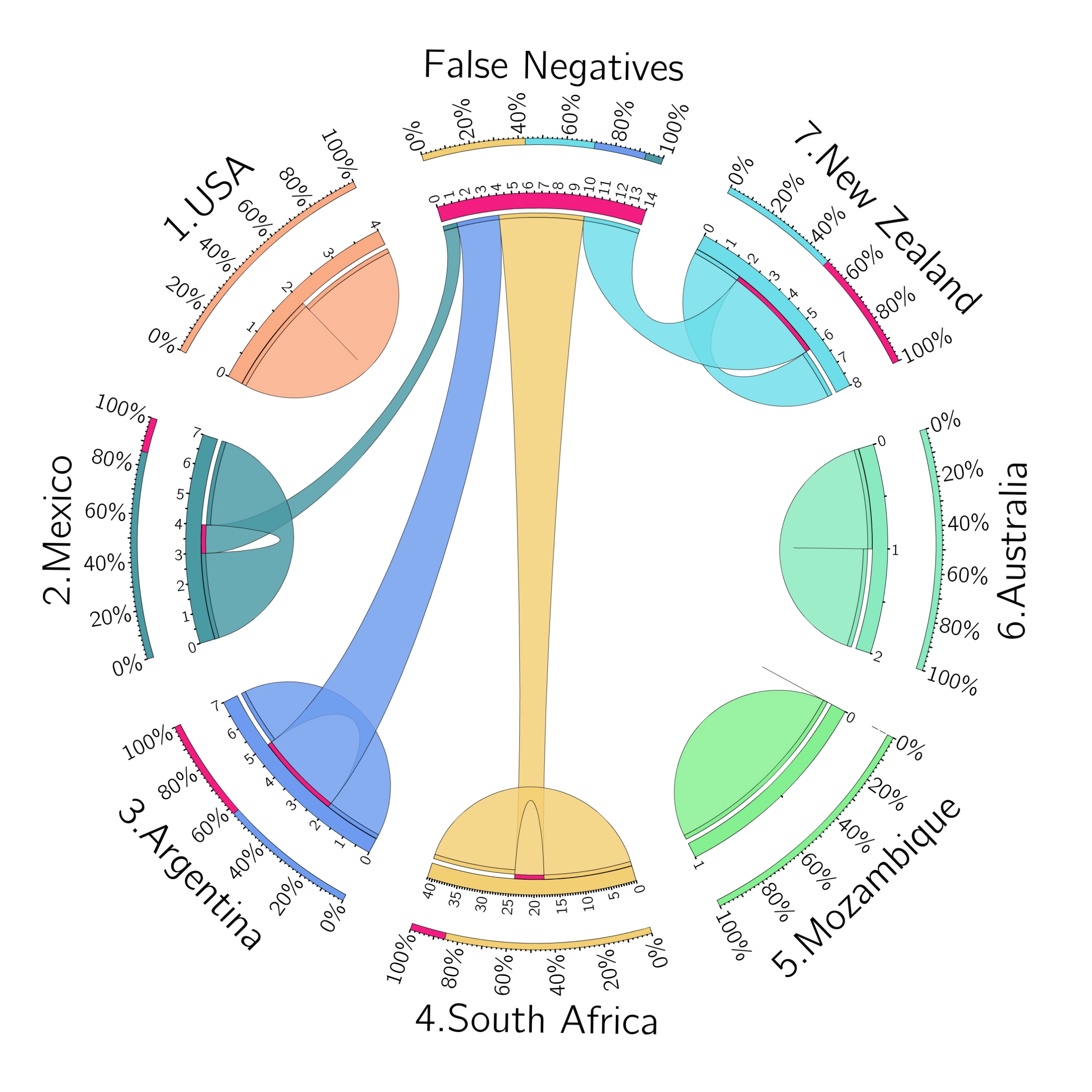


Fig. S1. Variation in the performance (false negatives) of the CNN-based model (step-1) across the six whale watching hotspots (only whale presence). Undetected whales in South Africa (42.85 % of FN), New Zealand (28.57 % of FN), Argentina (21.42 % of FN), and Mexico (7.14 % of FN), mainly because of lower spatial resolution, lower water-whale contrast, and greater frequency of submerged swimming posture. The presence of whales in Memba (Mozambique) was considered by the authors as uncertain due to the low resolution of Google Earth images (image source of SPOT-6 satellite).


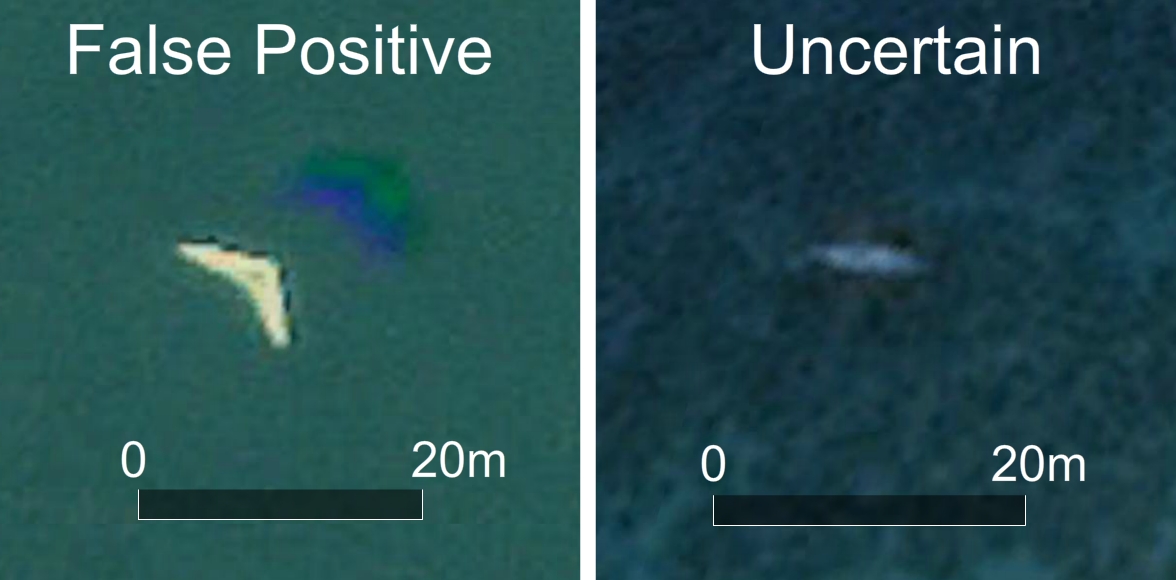


Fig. S2. An example of a false positive and a uncertain result. (Left) the figure shows a possible whale fluke in South Africa, which could be more likely a hang-glider. (Right) the image shows a possible whale but the authors defined it as uncertain due to low resolution of the image (SPOT-6 satellite). Map data: Google, DigitalGlobe


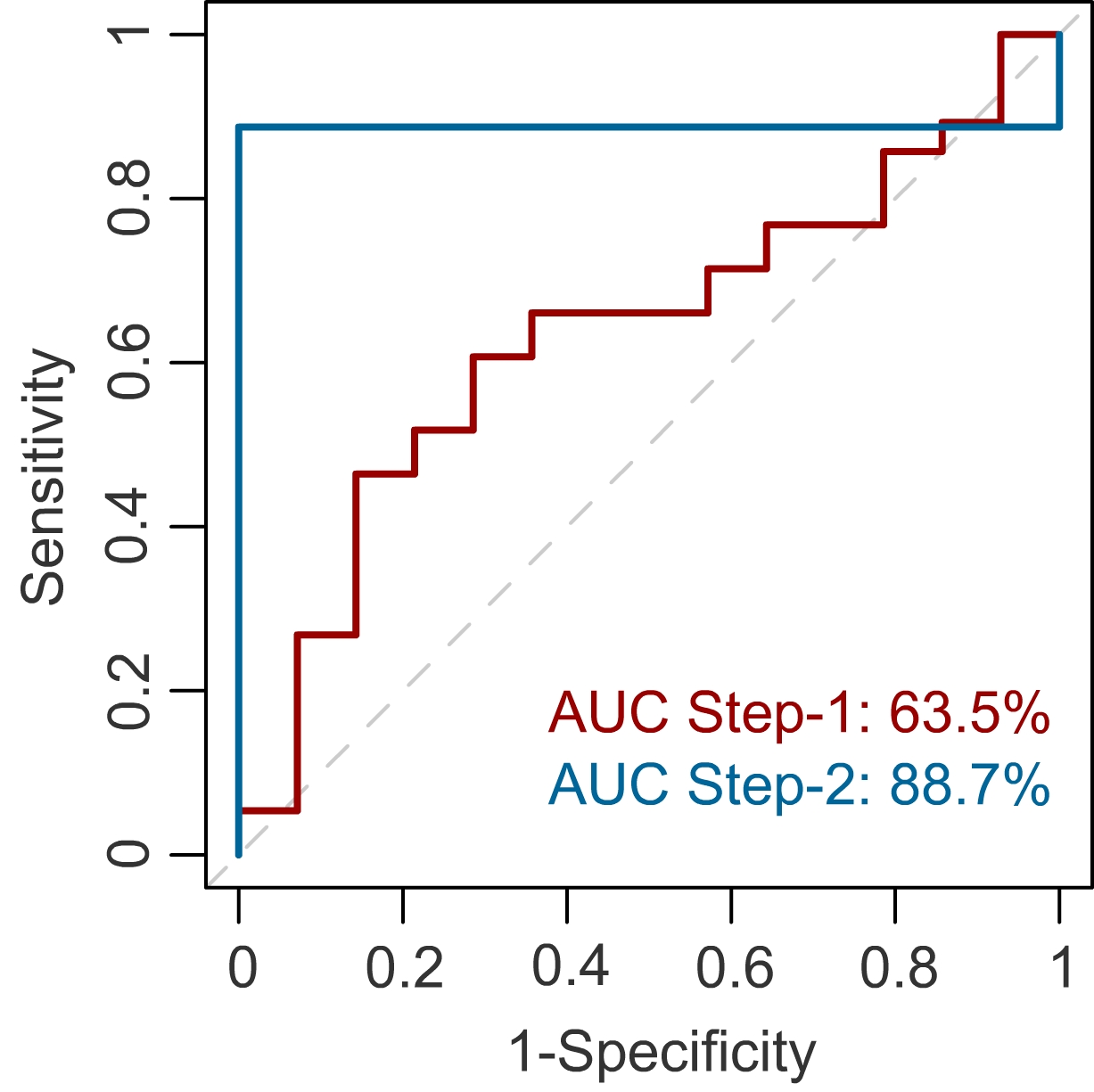


Fig. S3. Step-1(classification model) and step-2(detection model) performance with respect to ground truth.

**SUPPLEMENTARY DATA (Attached in a five csv files.)**

**Data S1. Metadata of the images for the class “Water + submerged rocks” in the training dataset.** For the 700 images extracted from Google Earth, it includes 1) the image ID, 2) latitude and 3) longitude coordinates (geographic WGS84) of the centroid of each 71 m × 71 m image cell, and 4) the photo-interpreted class label assigned by the authors (ground truth) .

**Data S2. Metadata of the images for the class “Ships” in the validation dataset.** For the 400 images extracted from Google Earth, it includes 1) the image ID, 2) latitude and 3) longitude coordinates (geographic WGS84) of the centroid of each 71 m × 71 m image cell, 4) the photo-interpreted class label assigned by the authors (ground truth), 5) the class predicted by the model, and 6) the maximum probability of the predicted class.

**Data S3. Metadata of the images for the class “Water + submerged rocks” in the validation dataset.** For the 400 images extracted from Google Earth, it includes 1) the image ID, 2) latitude and 3) longitude coordinates (geographic WGS84) of the centroid of each 71 m × 71 m image cell, 4) the photo-interpreted class label assigned by the authors (ground truth), 5) the class predicted by the model, and 6) the maximum probability of the predicted class.

**Data S4. Metadata of the images of the ten whale watching hotspots assessed in the validation dataset.** For the 13,348 images extracted from Google Earth, it includes 1) the image ID, 2) latitude and 3) longitude coordinates (geographic WGS84) of the centroid of each 71 m × 71 m image cell, 4) the photo-interpreted class label assigned by the authors (ground truth), 5) the class predicted by the model, 6) the maximum probability of the predicted class, and 7) the study site name (country).

**Data S5. Metadata of the results of the whale presence model (Step-1 CNN-based model) in the validation dataset.** For the 68 cell images extracted from Google Earth with photo-interpreted whale presence, it includes 1) the image ID, 2) latitude and 3) longitude coordinates (geographic WGS84) of the centroid of each 71 m × 71 m image cell, 4) the photo-interpreted class label assigned by the authors (ground truth), 5) the class predicted by the model, 6) the maximum probability of the predicted class, 7) swimming posture of whale photo-interpreted and 8) the study site name (country).
